# Loop‐mediated isothermal amplification for the detection of SARS‐CoV‐2 in saliva

**DOI:** 10.1111/1751-7915.13737

**Published:** 2021-01-26

**Authors:** Monika Janíková, Július Hodosy, Peter Boor, Boris Klempa, Peter Celec

**Affiliations:** ^1^ Institute of Molecular Biomedicine Faculty of Medicine Comenius University Bratislava Slovakia; ^2^ University Hospital Bratislava Slovakia; ^3^ Institute of Pathology Department of Nephrology University Clinic of the RWTH Aachen Germany; ^4^ Institute of Virology Biomedical Research Center Slovak Academy of Sciences Bratislava Slovakia; ^5^ Institute of Pathophysiology Faculty of Medicine Comenius University Bratislava Slovakia; ^6^ Department of Molecular Biology Faculty of Natural Sciences Comenius University Bratislava Slovakia

## Abstract

In the fight against the recent COVID‐19 pandemics, testing is crucial. Nasopharyngeal swabs and real‐time RT‐PCR are used for the detection of the viral RNA. The collection of saliva is non‐invasive, pain‐free and does not require trained personnel. An alternative to RT‐PCR is loop‐mediated isothermal amplification coupled with reverse transcription (RT‐LAMP) that is easy to perform, quick and does not require a thermal cycler. The aim of this study was to test whether SARS‐CoV‐2 RNA can be detected directly in saliva using RT‐LAMP. We have tested 16 primer mixes from the available literature in three rounds of sensitivity assays. The selected RT‐LAMP primer mix has a limit of detection of 6 copies of viral RNA per reaction in comparison with RT‐PCR with 1 copy per reaction. Whole saliva, as well as saliva collected using Salivette collection tubes, interfered with the RT‐LAMP analysis. Neither Chelex‐100 nor protease treatment of saliva prevented the inhibitory effect of saliva. With the addition of the ribonuclease inhibitor, the sensitivity of the RT‐LAMP assay was 12 copies per reaction of RNA in Salivette® saliva samples and 6 copies per reaction of RNA in whole saliva samples. This study shows that it is possible to combine the use of saliva and RT‐LAMP for SARS‐CoV‐2 RNA detection without RNA extraction which was confirmed on a small set of correctly diagnosed clinical samples. Further studies should prove whether this protocol is suitable for point of care testing in the clinical setting.

## Graphical abstract

We have tested 16 primer mixes for RT‐LAMP targeting SARS‐CoV‐2 RNA from the available literature in three rounds of sensitivity assays. The selected RT‐LAMP primer mix has a limit of detection of six copies of viral RNA per reaction. Whole saliva, as well as saliva collected using Salivette collection tubes interfered with the RT‐LAMP analysis, but this effect was prevented with the ribonuclease inhibitor, which was confirmed on a small set of correctly diagnosed clinical samples.

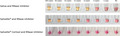

## Introduction

Testing and identification of infected persons is of utmost importance in the ongoing COVID‐19 pandemic (Sethuraman *et al*., 2020), especially due to the infectivity of asymptomatic or pre‐symptomatic carriers (Arons *et al*., 2020; Huff and Singh, 2020). Nasopharyngeal swabs and reverse transcriptase–polymerase chain reaction (RT‐PCR) are routinely used all over the world (Tang *et al*., 2020). Temporary shortage of chemicals for RNA isolation and PCR analysis, or even of swabs needed for sample collection indicated the need for other alternative diagnostic procedures (Esbin *et al*., 2020; Péré *et al*., 2020). Ideally, these should avoid the long logistic chain needed for sample collection, transport and processing in the laboratory (Rosenbaum, 2020).

Saliva is a diagnostic fluid with several key advantages (Kaczor‐Urbanowicz *et al*., 2017). Sample collection is easy to perform, non‐invasive, cheap, it does not require trained personnel and can, thus, be done at home or anywhere else. In addition, saliva represents a mixture of oral fluids from different sources (Proctor, 2016). This is of importance in comparison with swabs, which represent only a small part of the mucosal membranes in the nasopharyngeal or oropharyngeal cavities, which might be relevant for COVID‐19 diagnostics (Wyllie *et al*., 2020).

RT‐PCR is sensitive as well as specific and the method of choice for detecting specific sequences of nucleic acids, in this case the viral RNA (Corman *et al*., 2020). However, a thermal cycler with a fluorescence detection/camera is needed, the enzyme is easily affected by PCR inhibitors in the sample and is it currently not feasible to do the analysis as a bedside test (Tang *et al*., 2020). Loop‐mediated isothermal amplification coupled with reverse transcriptase (RT‐LAMP) is an alternative amplification method for detecting specific sequences of RNA (Wong *et al*., 2018). As the reaction is isothermal thanks to the activity of the Bst enzyme and the primer design, it does not require a thermal cycler or other specific equipment except a thermostat maintaining the temperature at 60‐65°C (Notomi *et al*., 2015). The outcome can be seen on gel electrophoresis, but also analysed with visual inspection using a pH‐sensitive dye (Goto *et al*., 2009; El‐Toloth et al., 2020) or fluorescence (Gonzalez‐Gonzalez et al., 2020). The simple procedure and outcome detection are of utmost importance for point of care testing (POCT) (Tomita *et al*., 2008).

Both saliva (Azzi *et al*., 2020; To *et al*., 2020) and RT‐LAMP (Baek *et al*., 2020; Yan *et al*., 2020; Yu *et al*., 2020; Huang et al., 2020, Lu et al.,2020b, Park et al., 2020, Zhang et al., 2020) have been used for SARS‐CoV‐2 detection. Saliva‐based tests have even been approved by the FDA for routine diagnostics, as the saliva analysis was found to be more consistent than nasopharyngeal swabs in direct comparison (Wyllie *et al*., 2020). However, most of the studies testing saliva used standard approaches for RNA isolation and RT‐PCR. These approaches would partially solve some issues with sampling, but not with the need for laboratory processing and analysis.

RT‐LAMP has been shown to be a feasible approach for testing the presence of SARS‐CoV‐2 RNA, but only after RNA isolation mostly from nasopharyngeal swabs. This would solve the issue with the needed infrastructure, that is real‐time PCR cyclers, but not the sampling‐related complications. One study reported the use of isolated salivary RNA as the template for RT‐LAMP (Rabe and Cepko, 2020). However, the RNA isolation procedure requires several steps that are difficult to perform as part of POCT. As it was shown that direct RT‐PCR from saliva is feasible, it should be possible to find conditions for RT‐LAMP directly from saliva. The aim of this study was to test whether SARS‐CoV‐2 RNA can be detected in saliva using RT‐LAMP.

## Results

### Selection of RT‐LAMP primer mixes

**Fig. 1:**
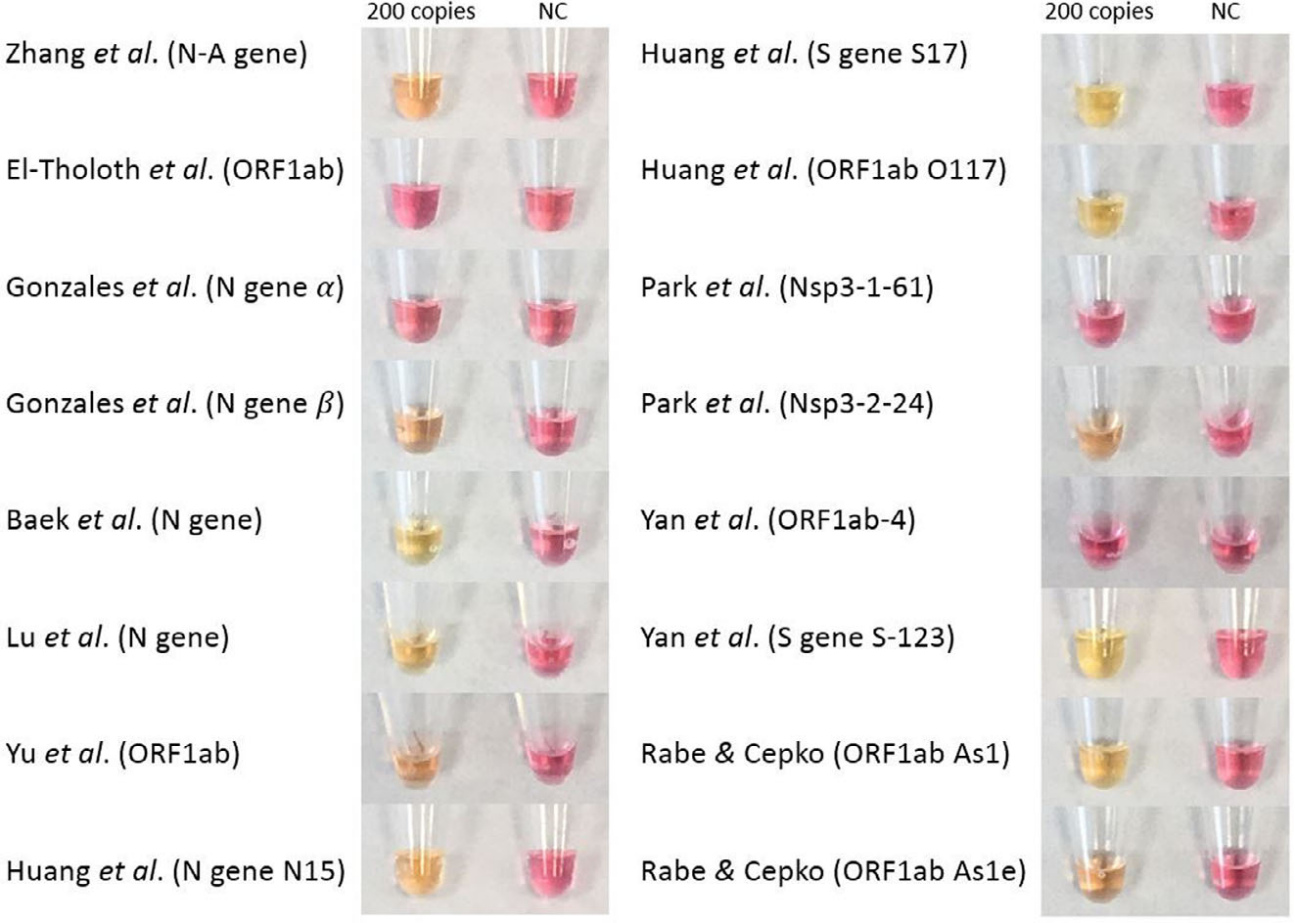
First screening of the 16 published RT‐LAMP primer mixes using 200 copies of the EDX SARS‐CoV‐2 RNA Standard. Yellow colour – positive result – presence of RNA, pink colour – negative result – absence of RNA. NC, negative control.

**Fig. 2:**
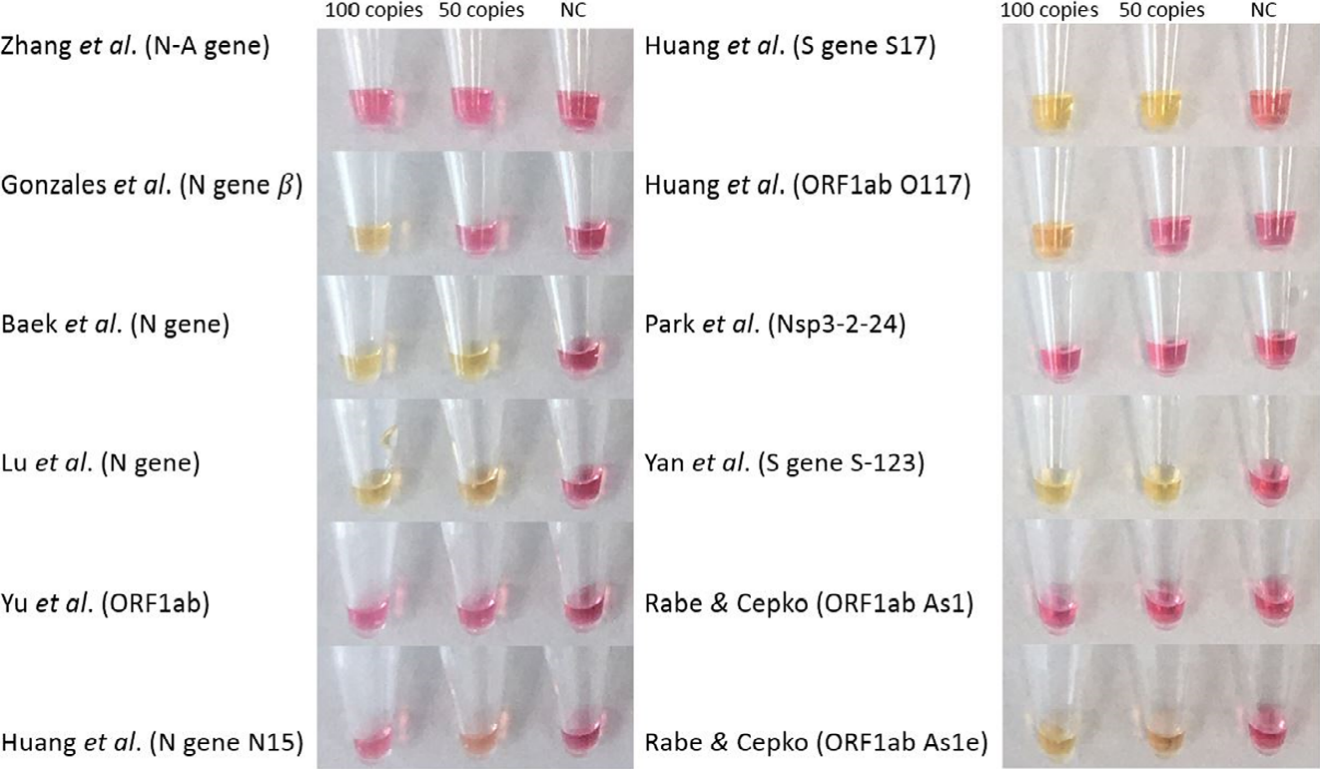
Second screening of the selected RT‐LAMP primer mixes using 100 and 50 copies of the EDX SARS‐CoV‐2 RNA Standard. Yellow colour – positive result – presence of RNA, pink colour– negative result – absence of RNA. NC, negative control.

**Fig. 3:**
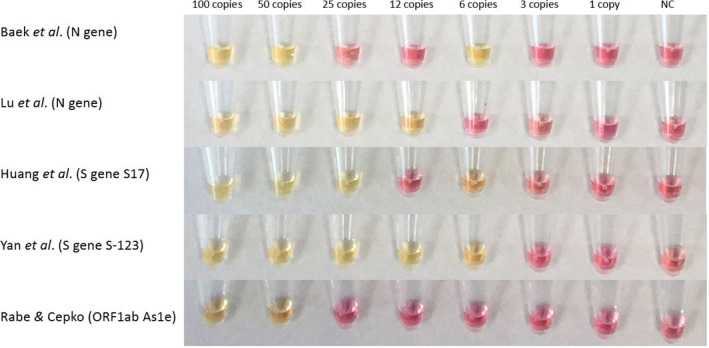
Sensitivity comparison of the RT‐LAMP primer mixes selected from the second screening using the EDX SARS‐CoV‐2 RNA Standard. Yellow colour – positive result – presence of RNA, pink colour – negative result – absence of RNA. NC, negative control.

We have chosen 16 primers sets for RT‐LAMP detection of SARS‐CoV‐2 targeting different genes and regions of the virus (ORF1ab, Nsp3, N gene, S gene) to evaluate the performance of these primers sets in three rounds. In the first screening, 16 primer mixes were tested in the RT‐LAMP reactions with 200 copies of the EDX SARS‐CoV‐2 RNA Standard per reaction. We assessed the outcome with naked eye after 30 min of incubation. Yellow colour of the reaction mixture represented a positive result – presence of RNA, pink colour indicated a negative result – absence of RNA (Fig. 1). All primer mixes were negative in the negative controls. Based on colour outcome, 12 primer mixes were chosen for the second screening. The procedure was repeated in the second screening with 12 primer mixes. The criteria for the next selection included a positive outcome for both 100 and 50 copies of the EDX SARS‐CoV‐2 RNA Standard per reaction. Only five out of 12 primer mixes met these criteria and were positive for 100 and 50 copies of the EDX SARS‐CoV‐2 RNA Standard (Fig. 2). Finally, in the third screening 5 primer mixes were tested for sensitivity using twofold diluted EDX SARS‐CoV‐2 RNA Standard from 100 copies to 1 copy per reaction. The primer mix designed and used by Yan *et al*. (S‐123) has repeatedly shown the lowest limit of detection at 6 copies of the EDX SARS‐CoV‐2 RNA Standard per reaction (Fig. 3).

### Colorimetric RT‐LAMP and Saliva samples

**Fig. 4:**
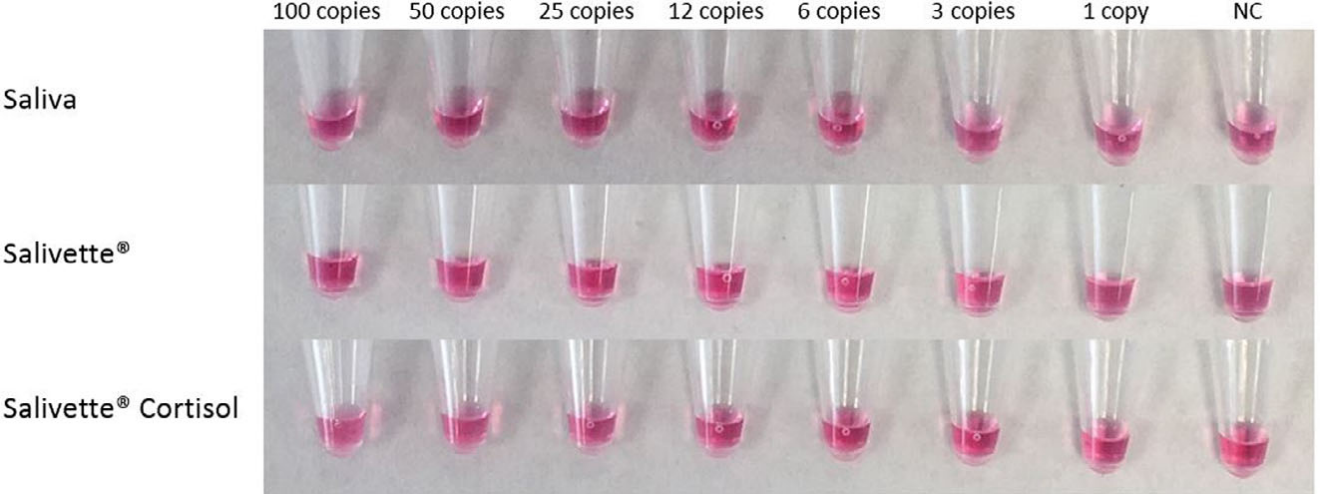
RT‐LAMP with untreated salivary samples with a serial dilution of the EDX SARS‐CoV‐2 RNA Standard as a RNA template using the selected Yan *et al*. (S‐123) primer mix. Yellow colour – positive result – presence of RNA, pink colour – negative result – absence of RNA. NC, negative control.

**Fig. 5:**
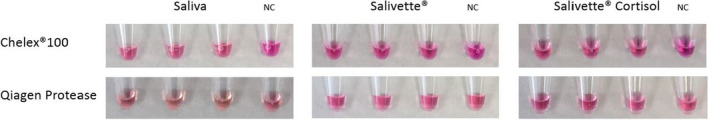
Effect of spiked saliva pretreatment on RT‐LAMP reactions performed with Yan *et al*. (S‐123) primer mix. Yellow colour – positive result – presence of RNA, pink colour – negative result – absence of RNA. NC, negative control.

**Fig. 6:**
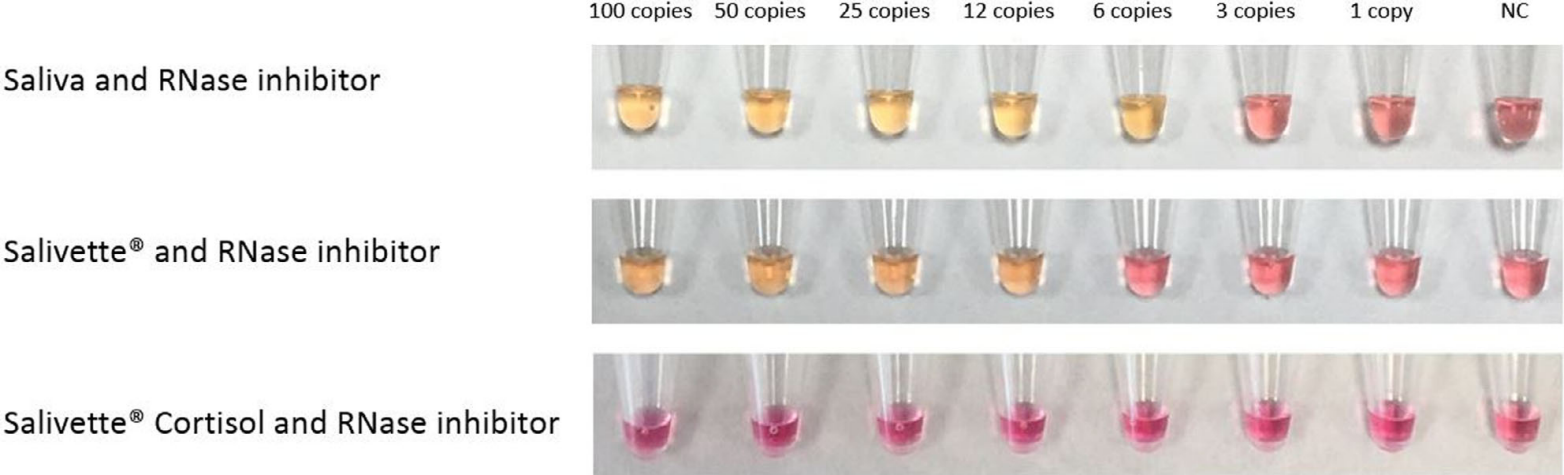
Effect of RNase inhibitor on RT‐LAMP on saliva samples with a serial dilution of the EDX SARS‐CoV‐2 RNA Standard as the RNA template performed with Yan *et al*. (S‐123) primer mix. Yellow colour – positive result – presence of RNA, pink colour – negative result – absence of RNA. NC, negative control.

Saliva samples were added to RT‐LAMP reaction mixtures. Based on previous results, the primer mix by Yan et al. (S‐123) was used. Twofold serial dilution of the EDX SARS‐CoV‐2 RNA Standard from 100 copies to 1 copy were added to the RT‐LAMP reaction as an RNA template. Saliva interfered with the RT‐LAMP reaction (Fig. 4), and we obtained a negative result. In an effort to improve the sensitivity, saliva samples spiked with the RNA were treated with Chelex®100 or Qiagen Protease. Neither Chelex®100, nor protease treatment of saliva samples helped to obtain the desirable result and all RT‐LAMP reactions were negative (Fig. 5). Finally, 8U of RNase inhibitor (Jena Bioscience, Jena, Germany) was added to the RT‐LAMP reactions to prevent the effects of RNases in saliva samples on EDX SARS‐CoV‐2 RNA Standard used as a RNA template. We obtained positive results for whole saliva samples where the limit of detection after addition of the RNase inhibitor was six copies of RNA per reaction and for Salivette® samples 12 copies of RNA per reaction (Fig. 6). In the case of Salivette® Cortisol, the obtained results remained negative (Fig. 6).

### Colorimetric RT‐LAMP and clinical saliva samples

**Fig. 7:**
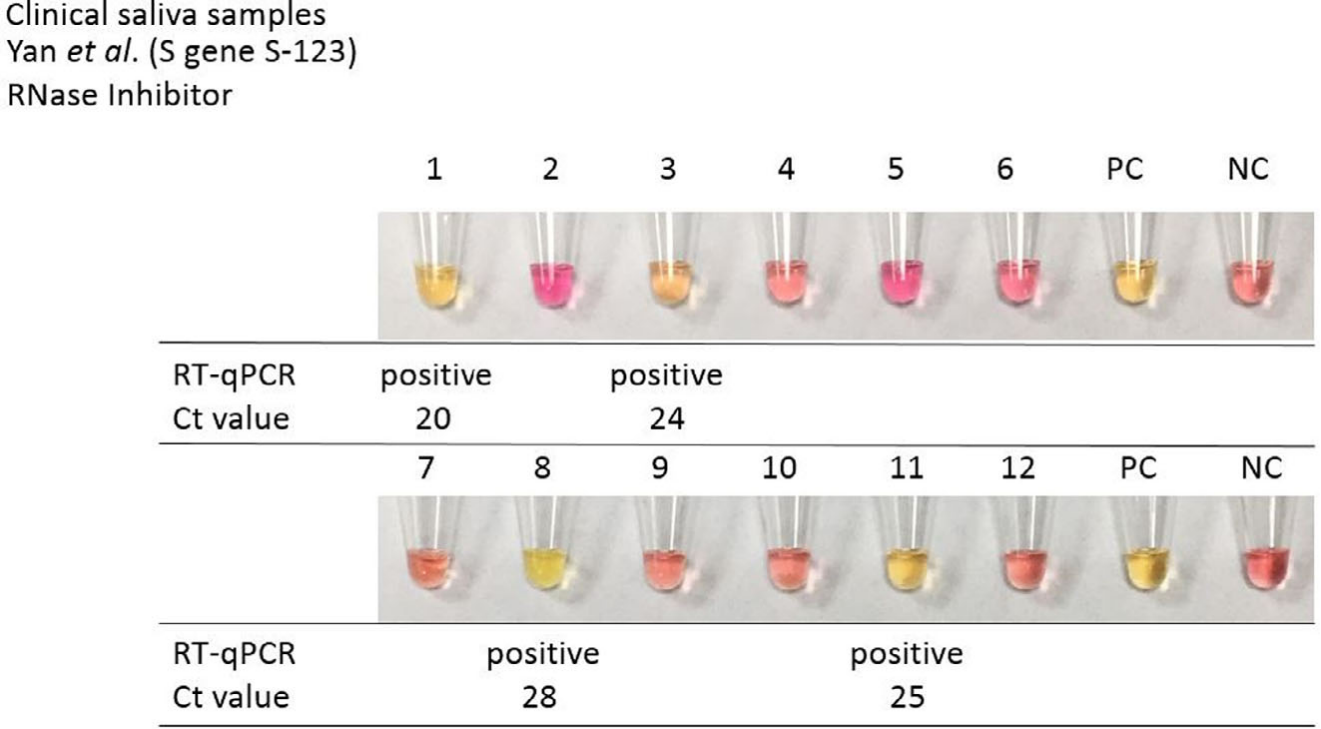
The RT‐LAMP on clinical saliva samples performed with Yan *et al*. (S‐123) primer mix and RNase inhibitor. Yellow colour – positive result – presence of RNA, pink colour – negative result – absence of RNA. PC, positive control; NC, negative control.

Clinical saliva samples were added to the RT‐LAMP reaction mixtures after thermal inactivation. Yan et al. (S‐123) primer mix was used in these RT‐LAMP reactions. RNase inhibitor (8U, Jena Bioscience, Jena, Germany) was added as we found that it improved detection of SARS‐CoV‐2 in saliva samples. In addition to clinical saliva samples (n = 10), we added spiked saliva samples with inactivated SARS‐CoV‐2 and control saliva samples to the RT‐LAMP reactions. From the clinical samples, three were positive and seven were negative (Fig. 7). Spiked saliva sample was positive, and the control saliva sample remained negative. RT‐LAMP results were compared with RT‐qPCR results from nasopharyngeal swabs. The clinical saliva samples positive in RT‐LAMP reaction were also positive in RT‐qPCR reaction. The remaining 7 samples were negative both in RT‐LAMP and RT‐PCR.

### One‐step real‐time RT‐PCR for SARS‐CoV‐2 sensitivity

**Fig. 8:**
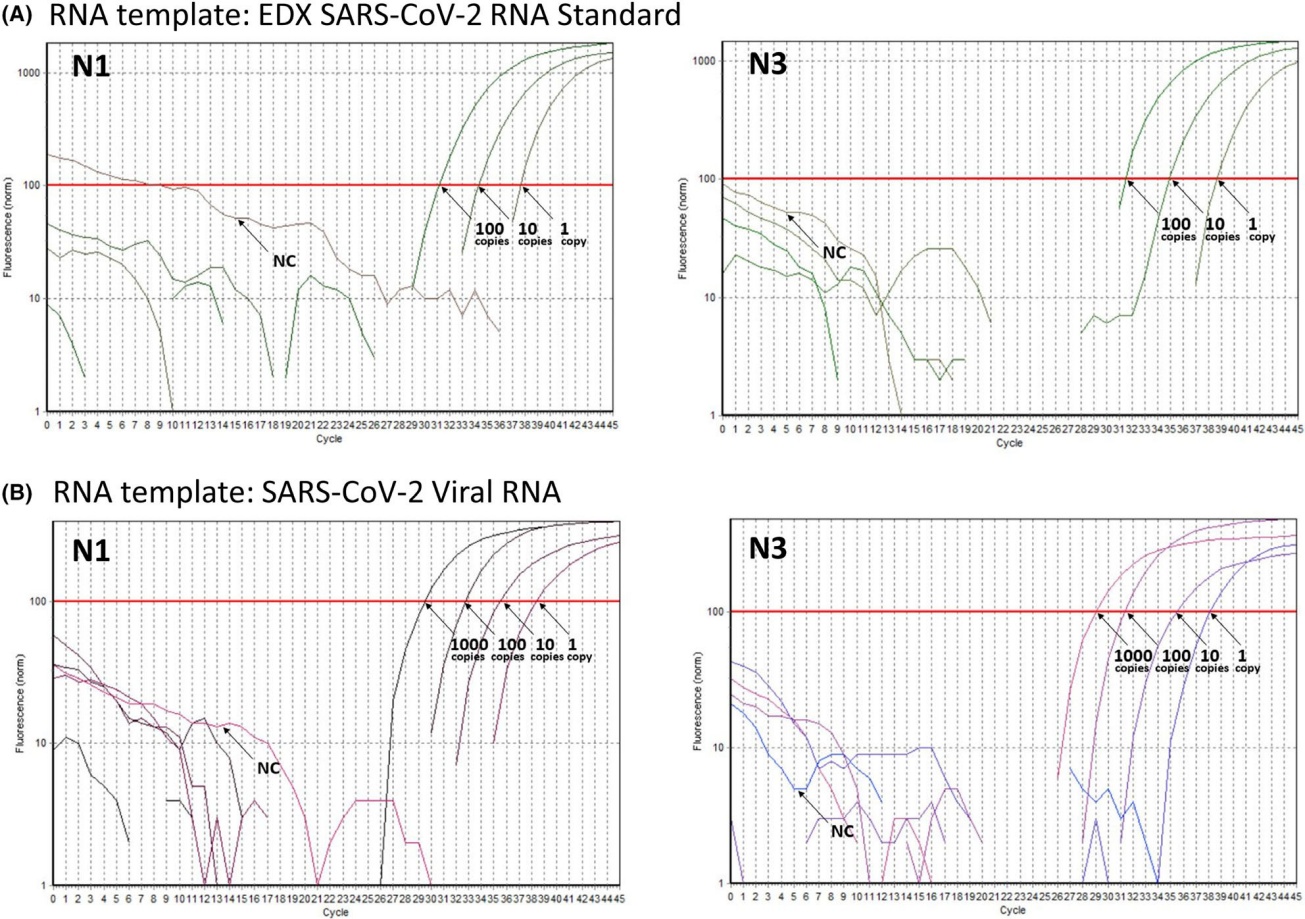
Sensitivity of real‐time RT‐PCR targeting N1 and N3 gene of the EDX SARS‐CoV‐2 RNA Standard – 100 copies, 10 copies and 1 copy (A). Sensitivity of real‐time RT‐PCR targeting N1 and N3 gene of the SARS‐CoV‐2 Viral RNA – 1000 copies, 100 copies, 10 copies and 1 copy (B). NC – negative control.

One‐step real‐time RT‐PCR with primers targeting N1 and N3 gene has shown that using this method it is possible to detect 1 copy of the EDX SARS‐CoV‐2 RNA Standard (Fig. 8A). Analysis of the isolated viral RNA from cell culture confirmed the same sensitivity (Fig. 8B). The specificity of the reaction was proved with a negative no template control after 45 cycles.

## Discussion

RT‐PCR on isolated RNA from nasopharyngeal swabs is the golden standard in the detection of SARS‐CoV‐2 (Corman *et al*., 2020; Wölfel *et al*., 2020). However, sampling, storage, transport and processing of samples especially due to the logistics takes 24 or even 48 h (Dramé *et al*., 2020). In the clinical practice, a rapid point of care diagnostics is often needed, ideally with a result within one or two hours. In this study, we have shown that the combination of using saliva as diagnostic fluid and RT‐LAMP as the analytical method is a feasible approach that can achieve very good sensitivity. Sensitivity of a diagnostic assay for COVID‐19 is a crucial analytical parameter, since false negativity is high even using standard procedures (Woloshin *et al*., 2020). As it depends on the localization and procedure of swab sampling, it is clear that the major issues are in the pre‐analytical part of the diagnostics (Lippi *et al*., 2020).

In direct comparison, saliva is more consistent in COVID‐19 diagnostics than swabs (Wyllie *et al*., 2020). In combination with the other advantages of non‐invasive sampling, saliva is likely the ideal diagnostic fluid for SARS‐CoV‐2 point of care detection (Han and Ivanovski, 2020; Xu *et al*., 2020). However, as seen on our results, even the addition of 1 μl of saliva can interfere with the RT‐LAMP reaction. Previously, Chelex®100 was successfully used to inactivate PCR inhibitors in saliva (Sweet *et al*., 1996; Polgárová *et al*., 2010). Pretreatment of saliva with Chelex®100, however, did not improve the outcome of RT‐LAMP in our study. Similarly, protease that could degrade interfering enzymes (Ben‐Assa *et al*., 2020) was not helpful in our hands. It is possible that the thermal inactivation of protease was not complete and the Bst polymerase in RT‐LAMP could be degraded. We have not analysed in detail the reasons why these pre‐treatments failed. We have rather tested a different approach based on the premise that saliva as a digestive fluid has strong nuclease and especially RNase activity (Robinovitch *et al*., 1968; Ceder *et al*., 1985). Indeed, the addition of the RNase inhibitor improved the sensitivity of the RT‐LAMP assay on spiked saliva samples. This indicates that the RNase activity of saliva is one of the major issues in salivary RNA diagnostics (Fábryová and Celec, 2014; Sullivan *et al*., 2020).

According to the published literature, clinical saliva samples from infective patients contain thousands and millions of viral particles per ml (Wölfel *et al*., 2020). The sensitivity of the assay with a limit of detection of 12 copies per reaction should, thus, be more than sufficient. To support this assumption, we have tested clinical saliva samples with the RT‐LAMP protocol and compared the outcome with RT‐qPCR results. Clinical saliva samples detected as positive in RT‐LAMP were positive also in RT‐qPCR with Ct values between 20 and 28. This is comparable to the results of a previously published study using a different protocol for saliva processing (Ben‐Assa *et al*., 2020). We did not observe any case of false positivity, but occasionally, during the optimization contamination of the negative control occurred. Paying attention to thorough cleaning of the working area, careful pipetting and discarding reaction tubes without opening seems to eliminate this issue. We have not tested the specificity of the assay on RNA from other coronaviruses, but this was tested in the original publication of the used RT‐LAMP primers (Yan *et al*., 2020). Although the incubation at 65°C for 30 min as part of the RT‐LAMP assay should inactivate any potential infective particles (Wang *et al*., 2020), when working with clinical samples we have included an inactivation step of 5 min at 95°C to avoid infections. Whether this step also increases the chances of RNA release and detection should be further investigated.

Salivette collection tubes represent an alternative to whole saliva collected by passive drooling (Lenander‐Lumikari *et al*., 1995). We have shown that their use affects the concentrations of selected salivary biomarkers (Kamodyová and Celec, 2011; Celec and Ostatníková, 2012). In this study, cotton and polyester Salivettes were tested. While saliva from both interfered with RT‐LAMP, RNase inhibitor was able to prevent the interference from saliva collected using cotton Salivette, but not using polyester‐based Salivette cortisol. Reasons might be related to the swab material and its interactions with the saliva content (Hansen *et al*., 2008; Durdiaková *et al*., 2012). However, based on the results passive drooling or cotton Salivette can be suggested for the collection of samples.

A major limitation of our study is that we have not tested the protocol on a large number of samples from COVID‐19 patients. Slovakia was very successful in preventing the infections in the first wave. We, thus, had to use the standard RNA and test the protocol on a limited set of samples. The virus will likely be more resistant to RNase, and thus, the sensitivity of the RT‐LAMP might be even higher. It cannot be ruled out that some of the less sensitive primer mixes might have a better performance with other reaction conditions on clinical samples (Liu *et al*., 2017). On the other hand, LAMP is relatively straightforward and unlike PCR, the reaction is not affected by subtle changes of conditions (Francois *et al*., 2011).

In conclusion, we have tested the currently published primer mixes for RT‐LAMP targeting SARS‐CoV‐2 RNA. We identified the most sensitive primer mix and used it for the analysis of spiked saliva samples. Successful detection of the SARS‐CoV‐2 RNA was possible after addition of the RNase inhibitor with very high sensitivity. While the method should be tested on a larger set of clinical samples, the combination of saliva and RT‐LAMP without the need for RNA isolation represents an easy, rapid and cost‐effective approach for point of care diagnostics in the COVID‐19 pandemics.

## Experimental procedures

### RT‐LAMP Primers

RT‐LAMP primers for the detection of SARS‐CoV‐2 targeting different genes and regions were selected from publications available at 1st May 2020 in PubMed or the preprint servers (Table S1). All primers for the 16 primer mixes were synthesized by Microsynth AG (Balgach, Switzerland).

### Colorimetric RT‐LAMP for SARS‐CoV‐2 primers screening

All equipment and PCR Box UVC/T‐AR (Biosan, Riga, Latvia) were sprayed with RNase Xterminator Spray (Grisp Research Solutions, Porto, Portugal) prior to experimental work. Dualfilter T.I.P.S.® (Eppendorf, Hamburg, Germany) was used to prevent contamination during the work. To avoid contamination from RT‐LAMP products, reaction tubes were not opened after incubation and evaluation. All experiments of RT‐LAMP were run repeatedly and in duplicates to verify interassay and intra‐assay reproducibility. Representative outcomes were photographed and are shown in the figures.

A 10× primer mix (FIP/BIP – 16 µM; F3/B3 – 2 µM; LF/LB – 4 µM) was mixed for all selected primer combinations (Table S1). Colorimetric RT‐LAMP reactions were performed with WarmStart® Colorimetric LAMP 2× Master Mix (DNA & RNA) (New England Biolabs, Ipswich, MA, USA). Preliminary, RT‐LAMP reaction volume of 25 µl was used. However, adjusted volume of 10 µl was found to be sufficient for a comparable performance in line with the manufacturer protocol (M1800). This lower volume might be important in times when chemicals are scarce. A 10 µl reaction mixture (WarmStart® Colorimetric LAMP 2x Master Mix DNA and RNA – 5 µl; 10× primer mix – 1 µl of the particular mix (Table S1); nuclease free water – 3 µl; RNA template – dilutions of SARS‐CoV‐2 RNA Standard – 1 µl) was gently mixed by pipetting and briefly centrifuged. Colorimetric RT‐LAMP assays were performed at 65°C for 30 min in an Eppendorf ThermoMixer C (Eppendorf, Hamburg, Germany).

A screening of 16 primer mixes was divided into 3 rounds. In the first screening, all 16 primer mixes were tested and 1 µl (200 copies) of the EDX SARS‐CoV‐2 RNA Standard was used as the RNA template for the RT‐LAMP reactions. 1 µl of nuclease free water was used as a negative control. The results of the colour reactions were evaluated with naked eye after a 30‐min incubation at 65°C.

The primer mixes with positive results for 200 copies of the EDX SARS‐CoV‐2 RNA Standard per reaction after the first screening (*n* = 12) were chosen for the second round of testing. In the second screening, 12 primer mixes were tested using 100 copies and 50 copies of the diluted EDX SARS‐CoV‐2 RNA Standard as the RNA template for the RT‐LAMP reactions. The results of the colour reactions were again evaluated with naked eye after a 30 min incubation at 65°C.

The primer mixes with positive results for both 100 and 50 copies of the EDX SARS‐CoV‐2 RNA Standard per reaction after the second screening (*n* = 5) were selected for the third round of testing. In the third screening, the sensitivity of five selected primer mixes was tested with twofold serial dilution from 100 copies to one copy of the EDX SARS‐CoV‐2 RNA Standard used as the RNA template for the RT‐LAMP reactions. The results of the colour reactions were again evaluated with naked eye after a 30 min incubation at 65°C. The most sensitive primer mix designed by Yan *et al*. (S‐123) was further used in combination with spiked saliva samples.

### Saliva collection and processing

Saliva samples were collected from healthy volunteers from the staff of the laboratory (*n* = 6). Whole saliva was collected by spitting into a sterile tube. Additional saliva samples were collected using Salivette® and Salivette® Cortisol (Sarstedt, Nümbrecht, Germany). The collected saliva samples were aliquoted. An aliquot of the samples was used for direct testing, and the rest was stored at −20 °C. Saliva was spiked with the EDX SARS‐CoV‐2 RNA Standard. Control and spiked samples (200 µl) were mixed with the Qiagen Protease (20 µl, 0.025 AU, Qiagen, Hilden, Germany), incubated at 56 °C for 10 min and heated to 70°C for 15 min to inactivate the protease. Alternatively, control and spiked saliva samples were mixed with 20 µl of 25% Chelex®100 solution (Sigma‐Aldrich, St. Louis, MO, USA) prepared with nuclease free water. The mixture was incubated at 56°C for 15 min and then heated in a microwave oven for 1 min. After incubation, the Chelex®100 particles in the mixture were allowed to settle to the bottom of the tube and the supernatant was further used. As another option, a ribonuclease (RNase) inhibitor (1:5, 8U, Jena Bioscience, Jena, Germany) was used during the preparation of the RT‐LAMP reactions with control and spiked saliva samples.

Nasopharyngeal swabs and saliva samples were collected at the University Hospital in Ružinov, Bratislava from patients suspected to be SARS‐CoV‐2 positive (*n* = 10). Nasopharyngeal swabs were collected by healthcare personnel and sent to a diagnostic laboratory for RT‐qPCR. Whole saliva was collected by spitting into a sterile tube and delivered to our laboratory. To avoid potential infections, but also as part of the sample processing, saliva samples were inactivated by heating at 95 °C for 5 min. Saliva samples were used for direct testing in RT‐LAMP as described above and aliquots were stored at −20 °C.

### Viral RNA extraction from cell culture supernatant

The SARS‐CoV‐2 viral RNA was isolated from cell culture supernatant obtained from Vero E6 cells infected with SARS‐CoV‐2 virus strain Slovakia/SK‐BMC5/2020 (https://www.european‐virus‐archive.com/virus/sars‐cov‐2‐strain‐slovakiask‐bmc52020‐fd) using QIAamp® Viral RNA Mini Kit (Qiagen, Hilden, Germany) following the spin protocol for purification of Viral RNA.

### One‐step real‐time RT‐PCR

For the real‐time RT‐PCR, we have used the US CDC SARS‐CoV‐2 primers (N1: fw‐GACCCCAAAATCAGCGAAAT, rev‐TCTGGTTACTGCCAGTTGAATCTG; N3: fw‐GGGAGCCTTGAATACACCAAAA, rev‐TGTAGCACGATTGCAGCATTG) synthesized by Microsynth AG (Balgach, Switzerland; Lu *et al*., 2020a). Each 10 µl reaction mix contained QuantiTect SYBR Green PCR Master Mix (Qiagen, Hilden, Germany) – 5 µl, 500 nM of forward and reverse PCR primers for N1 or N3 gene, QuantiTect RT Mix (Qiagen, Hilden, Germany) – 0.1 µl, RNA template – 1 µl (10‐fold dilutions of SARS‐CoV‐2 RNA Standard from 100 copies to 1 copy or SARS‐CoV‐2 viral RNA isolated from cell culture supernatant obtained from Vero E6 cells infected with SARS‐CoV‐2 virus strain Slovakia/SK‐BMC5/2020 and 2.9 µl nuclease free water. One‐step real‐time RT‐PCR was performed on an Eppendorf realplex^4^ Mastercycler epgradient S (Eppendorf, Hamburg, Germany) with thermocycling conditions set at 50 °C for 30 min for reverse transcription, 95 °C for 15 min for initial denaturation, followed by 45 cycles of amplification: 95 °C for 15 s, 55° for 30 s and 65 °C for 30 s.

## Conflict of interest

The authors have no conflict of interest to declare.

## Supporting information


**Table S1.** Sequences (5' to 3') of RT‐LAMP primers used in this study.
