# Supplementary material for: Loop‐mediated isothermal amplification for the detection of SARS‐CoV‐2 in saliva
Source: Microb Biotechnol. 2021 Jan 26;14(1):307–16. doi: 10.1111/1751-7915.13737 (PMC7888461; doi:10.1111/1751-7915.13737)
Supplement: Supplementary file 1 — Table S1. Sequences (5' to 3') of RT‐LAMP primers used in this study. [file MBT2-14-307-s001.docx]

**Supplementary Table 1.** Sequences (5' to 3') of RT-LAMP primers used in this study.

| Target | F3 | B3 | FIP | BIP | LF | LB | Citation |
| --- | --- | --- | --- | --- | --- | --- | --- |
| Gene N-A | TGGCTACTACCGAAGAGCT | TGCAGCATTGTTAGCAGGAT | TCTGGCCCAGTTCCTAGGTAGTCCAGACGAATTCGTGGTGG | AGACGGCATCATATGGGTTGCACGGGTGCCAATGTGATCT | GGACTGAGATCTTTCATTTTACCGT | ACTGAGGGAGCCTTGAATACA | Zhang *et al.*, 2020 (Zhang, et al., 2020) |
| ORF1ab | TGCTTCAGTCAGCTGATG | TTAAATTGTCATCTTCGTCCTT | TCAGTACTAGTGCCTGTGCCCACAATCGTTTTTAAACGGGT | TCGTATACAGGGCTTTTGACATCTATCTTGGAAGCGACAACAA | CTGCACTTACACCGCAA | GTAGCTGGTTTTGCTAAATTCC | El-Tholoth *et al.,* 2020 (El-Tholoth, et al., 2020) |
| N gene (α) | TGGACCCCAAAATCAGCG | GCCTTGTCCTCGAGGGAAT | CCACTGCGTTCTCCATTCTGGTAAATGCACCCCGCATTACG | CGCGATCAAAACAACGTCGGCCCTTGCCATGTTGAGTGAGA | TGAATCTGAGGGTCCACCAA | TTACCCAATAATACTGCGTCTTGGT | Gonzalez *et al.,* 2020 (Gonzalez-Gonzalez, et al., 2020) |
| N gene (β) | CCAGAATGGAGAACGCAGTG | CCGTCACCACCACGAATT | AGCGGTGAACCAAGACGCAGGGCGCGATCAAAACAACG | AATTCCCTCGAGGACAAGGCGAGCTCTTCGGTAGTAGCCAA | TTATTGGGTAAACCTTGGGGC | TAACACCAATAGCAGTCCAGATGA | Gonzalez *et al.,* 2020 (Gonzalez-Gonzalez, et al., 2020) |
| N gene | TGGACCCCAAAATCAGCG | AGCCAATTTGGTCATCTGGA | CGTTGTTTTGATCGCGCCCCATTACGTTTGGTGGACCCTC | ATACTGCGTCTTGGTTCACCGCATTGGAACGCCTTGTCCTC | TGCGTTCTCCATTCTGGTTACT | TCTCACTCAACATGGCAAGGAA | Baek *et al.,* 2020 (Baek, et al., 2020) |
| N gene | GCCAAAAGGCTTCTACGCA | TTGCTCTCAAGCTGGTTCAA | TCCCCTACTGCTGCCTGGAGCAGTCAAGCCTCTTCTCGTT | TCTCCTGCTAGAATGGCTGGCATCTGTCAAGCAGCAGCAAAG | - | TGGCGGTGATGCTGCTCTT | Lu *et al.,* 2020 (Lu, et al., 2020) |
| ORF1ab | CCACT  AGAGGAGCTACTGTA | TGACAAGCTACAACACGT | AGGTGAGGGTTTTCTACATCACTATATTGGAACAAGCAAATTCTATGG | ATGGGTTGGGATTATCCTAAA  TGTGTGCGAGCAAGAACAAGTG | CAGTTTTTAACATGTTGTGCCAACC | TAGAGCCATGCCTAA  CATGCT | Yu *et al.,* 2020 (Yu, et al., 2020) |
| N gene (N15) | AGATCACATTGGCACCCG | CCATTGCCAGCCATTCTAGC | TGCTCCCTTCTGCGTAGAAGCCAATGCTGCAATCGTGCTAC | GGCGGCAGTCAAGCCTCTTCCCTACTGCTGCCTGGAGTT | GCAATGTTGTTCCTTGAGGAAGTT | GTTCCTCATCACGTAGTCGCAACA | Huang *et al.,* 2020 (Huang, et al., 2020) |
| S gene (S17) | TCTTTCACACGTGGTGTT | GTACCAAAAATCCAGCCTC | CATGGAACCAAGTAACATTGGAAAACCTGACAAAGTTTTCAGATCC | CTCTGGGACCAATGGTACTAAGAGGACTTCTCAGTGGAAGCA | GAAAGGTAAGAACAAGTCCTGAGT | CTGTCCTACCATTTAATGATGGTGT | Huang *et al.,* 2020 (Huang, et al., 2020) |
| ORF1ab (O117) | CCCCAAAATGCTGTTGTT | TAGCACGTGGAACCCAAT | GGTTTTCAAGCCAGATTCATTATGGATGTCACAATTCAGAAGTAGGA | TCTTCGTAAGGGTGGTCGCAGCACACTTGTTATGGCAAC | TCGGCAAGACTATGCTCAGG | TTGCCTTTGGAGGCTGTGT | Huang *et al.,* 2020 (Huang, et al., 2020) |
| Nsp3  (1-61) | GGAATTTGGTGCCACTTC | CTATTCACTTCAATAGTCTGAACA | CTTGTTGACCAACAGTTTGTTGACTTCAACCTGAAGAAGAGCAA | CGGCAGTGAGGACAATCAGACACTGGTGTAAGTTCCATCTC | ATCATCATCTAACCAATCTTCTTC | TCAAACAATTGTTGAGGTTCAACC | Park *et al.,* 2020 (Park, et al., 2020) |
| Nsp3  (2-24) | TGCAACTAATAAAGCCACG | CGTCTTTCTGTATGGTAGGATT | TCTGACTTCAGTACATCAAACGAATAAATACCTGGTGTATACGTTGTC | GACGCGCAGGGAATGGATAATTCCACTACTTCTTCAGAGACT | TGTTTCAACTGGTTTTGTGCTCCA | TCTTGCCTGCGAAGATCTAAAAC | Park *et al.,* 2020 (Park, et al., 2020) |
| ORF1ab (4) | GGTATGATTTTGTAGAAAACCCA | CAACAGGAACTCCACTACC | GGCATCACAGAATTGTACTGTTTTTGCGTATACGCCAACTTAGG | AATGCTGGTATTGTTGGTGTACTGAGGTTTGTATGAAATCACCGAA | AACAAAGCTTGGCGTACACGTTCA | - | Yan *et al.,* 2020 (Yan, et al., 2020) |
| S gene (S-123) | TCTATTGCCATACCCACAA | GGTGTTTTGTAAATTTGTTTGAC | CATTCAGTTGAATCACCACAAATGTGTGTTACCACAGAAATTCTACC | GTTGCAATATGGCAGTTTTTGTACATTGGGTGTTTTTGTCTTGTT | ACTGATGTCTTGGTCATAGACACT | TAAACCGTGCTTTAACTGGAATAGC | Yan *et al.,* 2020 (Yan, et al., 2020) |
| ORF1ab (As1) | CGGTGGACAAATTGTCAC | CTTCTCTGGATTTAACACACTT | TCAGCACACAAAGCCAAAAATTTATCTGTGCAAAGGAAATTAAGGAG | TATTGGTGGAGCTAAACTTAAAGCCCTGTACAATCCCTTTGAGTG | TTACAAGCTTAAAGAATGTCTGAACACT | TTGAATTTAGGTGAAACATTTGTCACG | Rabe *&* Cepko, 2020 (Rabe and Cepko, 2020) |
| ORF1ab (As1e) | CGGTGGACAAATTGTCAC | CTTCTCTGGATTTAACACACTT | TCAGCACACAAAGCCAAAAATTTATTTTTCTGTGCAAAGGAAATTAAGGAG | TATTGGTGGAGCTAAACTTAAAGCCTTTTCTGTACAATCCCTTTGAGTG | TTACAAGCTTAAAGAATGTCTGAACACT | TTGAATTTAGGTGAAACATTTGTCACG | Rabe *&* Cepko, 2020 (Rabe and Cepko, 2020) |
